# Bio-derived carbon adsorbents from *Terminalia arjuna* bark for efficient bisphenol A removal: mechanistic insights toward sustainable water treatment

**DOI:** 10.1039/d5ra09019a

**Published:** 2026-01-22

**Authors:** A. S. Pathiraja, K. D. A. Dulanjana, G. Rajapaksa, P. W. Samarasekere

**Affiliations:** a Centre for Advanced Materials and Smart Manufacturing, Faculty of Computing and Technology, University of Kelaniya Kelaniya Sri Lanka pradeep.samarasekere@gmail.com; b Department of Zoology and Environmental Management, Faculty of Science, University of Kelaniya Kelaniya Sri Lanka

## Abstract

The development of sustainable sorbent materials capable of removing endocrine-disrupting contaminants from water is a pressing environmental challenge. In this study, bark powder of *Terminalia arjuna* (TABP), a traditional natural water purifying material, and its activated carbons (TAAC) were evaluated as bio-based adsorbents for the removal of bisphenol A (BPA), a pseudo-persistent and hazardous endocrine-disrupting chemical with significant risk to human health. Comprehensive characterization (FTIR, TGA, SEM-EDS, BET) revealed structural and chemical transformations during activation, including enhanced aromaticity and reduced oxygenated groups. Batch adsorption studies showed that bark material exhibited higher capacity (36.10 mg g^−1^), while activated charcoal derived at 500 °C achieved faster uptake (>95% removal in 30 min) with strong affinity (*R*_L_ = 0.018). Isotherm analysis indicated that the combined Langmuir-Hill behaviour reflects site-limited adsorption with cooperative effects, consistent with dominant specific interactions rather than purely surface-area-driven physisorption. The Temkin model further suggested significant adsorbate–adsorbent interactions dominated by chemisorption. Kinetic analysis showed excellent agreement with the pseudo-second-order model (*R*^2^ > 0.998), supporting chemisorption as the rate-limiting step. These findings highlight the potential of *Terminalia arjuna*–derived adsorbents as promising precursors for developing efficient, renewable carbon adsorbents with well-defined molecular-level interactions. By coupling sustainability with mechanistic understanding, this work highlights design principles that can inform the development of next-generation sorbents for contaminant removal in resource-limited, and environmentally sensitive water treatment applications.

## Abstract 2

The development of sustainable sorbent materials capable of removing endocrine-disrupting contaminants from water is a pressing environmental challenge addressed here using KOH-activated carbon from *Terminalia arjuna* bark for efficient BPA removal.

## Graphical abstract

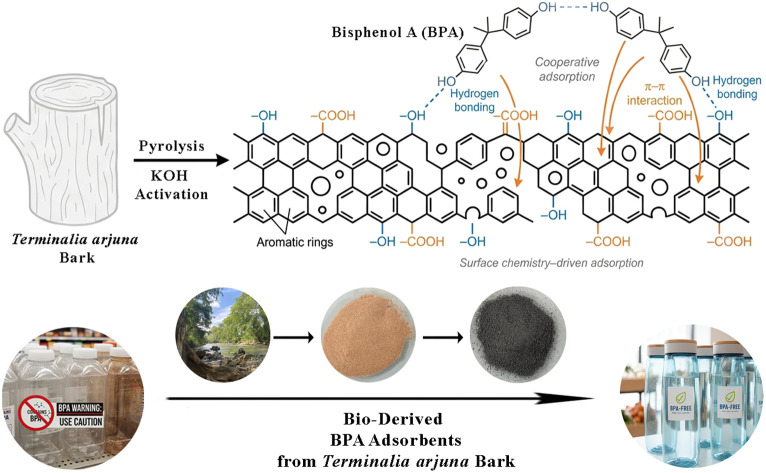

## Introduction

Rapid urbanization and industrial expansion have significantly intensified the concerns over safety and purity of drinking water. Among various water pollutants, bisphenol A [4,4′-(propane-2,2-diyl) diphenol] commonly abbreviated as BPA is a prevalent endocrine-disrupting chemical (EDC) worldwide. BPA is primarily used in the production of polycarbonate plastics and epoxy resins,^1^ which are widely found in consumer products. BPA is frequently detected in environmental matrices such as surface water, ground water, and drinking water in the concentration levels from nanograms to micrograms per litter.^2,3^ Human exposure to BPA occurs primarily through ingestion of BPA-contaminated food and water, while occupational exposure, inhalation, and dermal absorption have also been shown to occur. BPA exhibits endocrine-disrupting activity in humans by binding to several endocrine receptors and numerous studies have linked long-term BPA exposure, even at trace levels, to adverse health outcomes including various forms of cancer.^4–6^

Due to its widespread distribution and potential health risks, there is a pressing need for efficient techniques to remove BPA from water systems. To address this challenge, numerous BPA remediation technologies have been employed. Among them, adsorption using bio-based natural materials, such as activated carbon derived from agricultural or lignocellulosic waste has gained attention as an effective, economical, and environmentally benign approach.^7,8^ Biomass-derived activated carbon offers a range of advantages including high surface area, well-developed pore structures of micro, meso, and macropores, abundant functional groups, and ease of regeneration.^9^ The functional moieties such as carboxyl, carbonyl, phenol, lactone, and quinone groups, contribute to enhanced interactions with a wide range of organic pollutants, including hydrophobic and hydrophilic compounds.^7,10^

In a study conducted by Lazim *et al.*, coir pith, durian peel, and coconut shell were used to remove BPA with removal efficiencies of 72%, 70%, and 69%, and adsorption capacities of 4.308 mg g^−1^, 4.178 mg g^−1^, and 4.159 mg g^−1^, respectively.^11^ Senol *et al.* has experimented with waste coffee for BPA removal with the maximum removal percentage of 86%.^12^ According to Hayoun *et al.* 2021, sunflower seed shells have high adsorption capacity of 22.54 mg g^−1^ with low removal efficiencies of about 20%.^13^ In a study conducted by Ahsan *et al.*, Tea leaves were used to remove BPA, and it showed removal efficiency about 60%.^14^ In consecutive studies, Lazim *et al.*, identified durian peel, banana bunch, and coconut bunch as absorbents to remove BPA with the removal efficiencies of 66%, 75%, and 76%, and adsorption capacities of 4.178 mg g^−1^, 4.53 mg g^−1^, and 4.66 mg g^−1^ respectively.^15,16^ Arampatzidou and Deliyanni achieved an exceptionally high adsorption capacity of 454.62 mg g^−1^ using potato peels-derived activated carbon.^17^ Palm shell (62.5 mg g^−1^), milk vetch (33.2 mg g^−1^), *Ulva prolifera* (90%, 33.3 mg g^−1^), palm kernel shell (90%, 37.8 mg g^−1^), and waste coffee grounds (123.2 mg g^−1^) have also shown promise as BPA adsorbents.^18–21^ The diversity in performance of BPA adsorption is largely attributed to differences in surface functionality, pore structure, and synthesis methods.

Despite extensive research on BPA adsorption using biochar and activated carbon materials, most studies primarily emphasize maximizing surface area and adsorption capacity, often with limited consideration of how surface chemical functionality governs adsorption behaviour. Adsorption systems exhibiting high removal efficiency despite low surface area remain poorly understood, and mechanistic interpretations are frequently inferred without sufficient correlation to material chemistry. Addressing this gap is essential for developing functionally optimized, sustainable bio-based adsorbents beyond conventional surface-area-driven design strategies. Regardless of the advances in high-porosity activated carbons, the gaps also remain in low-cost, minimally processed bio-adsorbents derived from traditional medicinal plants. This study draws on the traditional ecological knowledge, highlighting how centuries-old natural remediation practices, such as the use of *Terminalia arjuna*, can be adapted to address modern environmental pollutants like BPA. *Terminalia arjuna* bark, rich in bioactive compounds including terpenoids, flavonoids, polyphenols, steroids, glycosides, tannins, arjunic acid, terminic acid, and oligomeric proanthocyanidins with known affinity for aromatic compounds, has been used traditionally for water purification in South Asia.^22^ It's chemically rich lignocellulosic composition offers diverse oxygen-containing functional groups relevant for investigating surface chemistry-dominated adsorption mechanisms.^23^ This makes *Terminalia arjuna* a suitable model precursor for examining adsorption behaviour where contribution of surface chemical interactions, accessible functional groups, and pore accessibility may play a more critical role than total surface area. BPA biosorption generally involves binding of BPA molecule to polar functional groups such as hydroxyl and carbonyl groups, making it a promising precursor for biosorbent synthesis. Previous studies have employed *Terminalia arjuna*-derived materials for the removal of dyes, heavy metals, and phenolic compounds from water. For instance, *Terminalia arjuna* sawdust waste can be used to remove crystal violet dye with an adsorption capacity of 46.0 mg g^−1^ within 120 minutes.^24^*Terminalia arjuna* fruit powder was employed for lead removal, leaves were used for the zinc removal, and bark powder facilitated the rare earth element-praseodymium biosorption.^23,25,26^ However, to the best of our knowledge, no studies have explored the use of *Terminalia arjuna* for the adsorption of BPA from aqueous systems. This study aims to evaluate the adsorption potential of *Terminalia arjuna* bark powder (referred as TABP) and mildly KOH-modified pyrolyzed bark powder (referred as TAAC) for BPA removal under varying environmental conditions, and by characterizing the adsorbent through a suite of analytical techniques. These materials are rooted in centuries-old traditional water purification practices used in rural communities. By re-examining these approaches in the context of contemporary environmental challenges, this study demonstrates that time-tested techniques can also provide effective treatment for pollutants characteristic of modern industrial and urban discharges. Future work will validate performance in natural water matrices to account for competing species and environmental complexity.

## Materials and methods

### Materials

All chemicals were of analytical grade and used without further purification. BPA was obtained from HiMedia Laboratories, LLC, India. Selected physiochemical properties of BPA are presented in Table 1. Ultrapure water was used for the preparation of all solutions. BPA solutions were freshly prepared prior to each experiment to minimize photodegradation and microbial degradation. The following analytical-grade reagents were used: potassium hydroxide (KOH, 88.2%, VWR, BDH chemical, Belgium), methanol (CH_3_OH, 99.8%, LOBA Chemie Pvt. Ltd, India), sodium hydroxide (NaOH, 98%, Research-Lab Fine Chem Industries, India), and hydrochloric acid (HCl, 37%, Fisher Scientific, UK). Acetonitrile was obtained in HPLC grade (C_2_H_3_N, VWR Chemicals BDH, France).

**Table 1:** Chemical properties of bisphenol A (BPA)

Compound name	Molecular formula	p*K*_a_	Molecular Mass	Chemical structure
Bisphenol A	C_15_H_16_O_2_	9.59	228 g mol^−1^	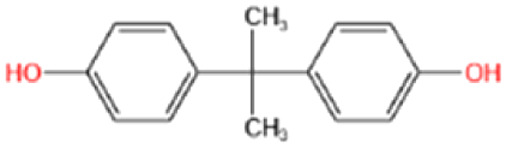

### Raw materials and activated carbon preparation

Barks of *Terminalia arjuna* was locally sourced in Sri Lanka. The bark was rinsed with distilled water to remove surface impurities, air-dried for 48 hours, cut into small pieces, ground using a mechanical grinder, and sieved to a particle size of <40 µm (250 mesh). The prepared biomass was stored in airtight containers until further use. The KOH-modified carbonaceous adsorbent was prepared following previously reported protocols with minor modifications rather than in-study optimization.^27,28^ The dried and sieved TABP was chemically activated in a singlestep process using 1 M KOH at a 1 : 2.5 weight-to-volume (w/v) ratio. The mixture was left overnight for thorough impregnation, then oven-dried at 105 °C to constant weight. Supong *et al.* (2019) reported that activation of biomass using 1 M KOH with an impregnation ratio of approximately 1 : 2 (w/v) provided effective activation for BPA adsorption while preserving surface reactivity.^28^ Accordingly, a comparable ratio (1 : 2.5, w/v) was employed in this study to ensure sufficient chemical modification without excessive degradation of surface functional groups. Although the material is chemically modified using KOH, it was observed to have a relatively low surface area, indicating limited pore development. Therefore, the term ‘activated carbon’ is used descriptively rather than to imply conventional high-surface-area commercial activated carbons. The KOH-loaded samples were carbonized in a tube furnace under a nitrogen atmosphere with a flow rate of 5 L min^−1^ at target temperatures of 500 °C and 800 °C for 1 hour. The resulting carbons were washed with 0.1 M HCl and distilled water until a neutral pH was achieved, then oven-dried and stored in sealed glass bottles. The KOH-modified carbonaceous adsorbent products are referred to as TAAC-500 and TAAC-800, respectively.

### Batch adsorption experiments

A 1000 mg L^−1^ stock solution of BPA was prepared in a 5% methanol–95% ultrapure water mixture to improve BPA solubility. Working solutions were prepared by serial dilution of the stock. The methanol concentration was kept constant across all experiments and is sufficiently low to minimize competitive adsorption or alteration of adsorption mechanisms, serving solely as a solubilizing agent for BPA. Similar experimental approaches have been widely adopted in BPA adsorption studies.^11,14,15^ Batch adsorption experiments were performed using both TABP and TAAC in 150 ml Erlenmeyer flasks containing 50 ml BPA solutions with varying concentrations and adsorbent dosages. The flasks were covered with aluminium foil to prevent photodegradation. The mixtures were agitated at 100 rpm at room temperature in an orbital shaker for 24 hours to achieve equilibrium. The supernatants were collected by filtration, and residual BPA concentrations were determined by high-performance liquid chromatography (HPLC). Control experiments without the adsorbent confirmed negligible BPA loss in the absence of sorbent materials. The effect of adsorbent dosage on BPA removal was evaluated using a BPA solution with the initial concentration of 500 mg L^−1^, with varying masses of TABP and TAAC in the range of 0.3 to 0.8 g. The experiment dosages were added to flasks which were subsequently agitated for 24 hours to achieve equilibrium. To determine the equilibrium time and adsorption kinetics, time-dependent studies were carried out using 50 mL of 500 mg L^−1^ BPA solutions and optimized dosage of TABP (0.6 g) and TAAC-500 (0.7 g) as determined from the dosage experiments. The samples were withdrawn at predetermined intervals in the range of 5 to 480 minutes, centrifuged, and filtered prior to quantification of residual BPA by HPLC. The effect of pH in BPA adsorption was assessed across the pH range 4 to 10. The pH was adjusted using 0.1 M NaOH or 0.1 M HCl and verified with a calibrated pH meter. The experiments were conducted using 50 ml of 500 mg L^−1^ BPA solutions and optimized dosage of adsorbents. After 24 hours of agitation, residual BPA concentrations were measured to evaluate the adsorption performances.

### Analytical methods

BPA concentrations were quantified using high-performance liquid chromatography on a Nexera LC-40 XR Modular HPLC (Shimadzu Corporation, Kyoto, Japan), equipped with 4.6 mm x 150 mm Restek C18 column (Restek Corporation, Bellefonte, PA, USA) and a PDA (photodiode array) detector. The mobile phase consisted of 55% acetonitrile and 45% ultrapure water, with a flow rate of 0.8 mL min^−1^. Injection volume was 20 µL, whereas, run time and detection wavelength were adjusted to 7 minutes and 254 nm. All samples were filtered through 0.45 µm membrane filters prior to injection. pH was measured at room temperature using Eutech pH 700 bench-top pH meter with ATC probe (Oakton Instruments, Vernon Hills, IL, USA). All measurements were performed in triplicate, and results were reported as mean ± standard deviations.

### Characterization of adsorbents

The surface functional groups of TABP and TAAC, before and after BPA adsorption, were characterized using Fourier-transform infrared spectroscopy (FT-IR) on a Nicolet iS10 spectrometer (Thermo Fisher Scientific Inc., Madison, WI, USA). Measurements were carried out in the infrared range of 4000–400 cm^−1^ using an attenuated total reflectance (ATR) equipped with a ZnSe crystal. All spectra were baseline-corrected against an air background, with a fresh reference air spectrum collected after each scan. The resulting spectra were recorded as absorbance values at each data point, and each measurement was performed in triplicate. Thermal stability of the adsorbents was assessed *via* thermogravimetric analysis (TGA) on a Discovery SDT 650 analyzer (TA Instruments, New Castle, DE, USA). Measurements were carried out from room temperature to 800 °C at a heating rate of 10 °C min^−1^ under a nitrogen atmosphere. Surface morphology and elemental composition were determined using field-emission scanning electron microscopy (FE-SEM) integrated with energy-dispersive X-ray spectroscopy (EDX) on a Hitachi Su6600 FE-SEM (Hitachi High-Technologies Inc., Tokyo, Japan) and an Oxford Instruments EDX (Oxford Instruments NanoAnalysis, High Wycombe, UK) with AZtec software (Version 6.1), with samples that were gold-coated prior to imaging. EDX analysis was conducted at an accelerating voltage of 15 kV, for reliable detection and mapping of the elemental composition on the TABP and TAAC surfaces. Textural properties, including pore volume, pore size distribution and specific surface area, were determined using the Brunauer–Emmett–Teller (BET) method by using Autosorb iQ Analyzer (Quantachrome Instruments, Boynton Beach, FL, USA), after degassing at a temperature of 300 °C.

### Point of zero charge (pH_pzc_) and effect of ionic strength determination

The point of zero charge (pH_pzc_) of the adsorbent was determined using the salt-addition method. In this procedure, 50 ml of 0.01 M NaCl was distributed into a series of flasks, and the initial pH was adjusted to 4 to 10. A constant mass of adsorbent (0.6 g) was added to each flask and equilibrated for 24 hours. The final pH values were recorded, and the pH_pzc_ was identified as the point where the curve of final pH *versus* initial pH intersected the line pH_initial_ = pH_final_. Further experiments were conducted to assess the influence of background electrolytes on BPA adsorption, whereas batch experiments were performed with NaCl concentrations of 0.02 M and 0.04 M under optimized adsorption conditions.

### Adsorption isotherms and kinetics

Adsorption isotherm studies were performed by equilibrating optimized adsorbent dosages with varying initial BPA concentrations at controlled pH, temperature, and agitation conditions. After 24 h equilibration, equilibrium concentrations were measured, and the adsorption capacities were calculated. This data was used to standardize the amount of BPA adsorbed per unit mass of adsorbent and to compare different adsorption models. To describe the adsorption behaviour, equilibrium data were analyzed using Langmuir, Freundlich, Temkin, Hill, and Jovanovich isotherm models. Kinetic studies were conducted to determine the rate of BPA uptake and mass transfer rate by fitting the kinetic data to pseudo-first-order and pseudo-second-order models. Nonlinear regression analyses were performed using OriginPro 2022 software to obtain model parameters.

All adsorption experiments were conducted in triplicate, and the reported values represent the mean ± standard deviation. Some of the small standard deviations observed across replicate experiments indicate good reproducibility and support the reliability of the reported adsorption trends.

## Results and discussion

The material characterization results are discussed in direct relation to adsorption performance, emphasizing how activation-induced changes in surface chemistry influence BPA adsorption behaviour.

### Characterization of adsorbent

**Fig. 1:**
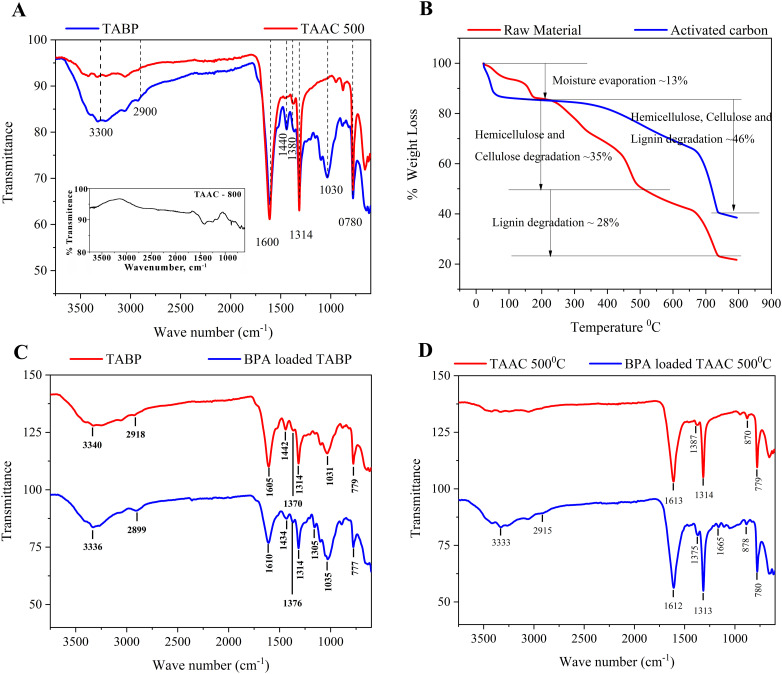
(A) FTIR spectra of TABP, TAAC-500, and TAAC-800 (inset) showing progressive loss of hydroxyl, aliphatic, and oxygenated functionalities upon activation; (B) TGA thermograms of TABP and TAAC-500 illustrating the thermal decomposition of hemicellulose, cellulose, and lignin; (C and D) FTIR spectra before and after BPA adsorption for TABP and TAAC-500 highlighting enhanced O–H stretching and shifts in aromatic C=C bands, consistent with hydrogen bonding and π–π interactions as dominant adsorption mechanisms.

FT-IR spectroscopy provided insights into the surface functional groups of raw bark powder (TABP) and the derived activated carbon (TAAC) (Fig. 1A). Distinct spectral differences were observed between the raw and pyrolyzed materials. A broad, intense band at 3340 cm^−1^ in TABP corresponds to the –OH group stretching of aliphatic and aromatic hydroxyl groups and adsorbed water molecules.^29^ The substantial reduction of this band in TAAC indicated dehydration during the activation. Similarly, the peak observed at 2918 cm^−1^ in TABP, associated with C–H stretching in CH_2_ groups decreased after converting to activated carbon, reflecting the loss of polar functional group.^29^ The sharp intense bands at 1605 and 1616 cm^−1^ in TABP and TAAC-500 were assigned to C=O stretching of lactone/carbonyl groups, C=C stretching lactone/carbonyl groups, and C=C stretching of alkene or aromatic ring, while these bands were absent in TAAC-800, suggesting progressive decomposition at higher pyrolysis temperatures.^30^ Further, skeletal C=C vibrations of aromatic rings are indicated by the peaks at 1448 cm^−1^ in TABP.^17^ Peaks at 1370 and 1387 cm^−1^ were attributed to C–H bending vibration or C–N stretching vibrations, while the 1314 cm^−1^ in TABP and TAAC-500 indicated phenolic C–O stretching mode.^30,31^ A broad band around 1031 cm^−1^, assigned to C–O stretching in the methoxy group (–OCH_3_), diminishes in TAAC samples again highlighting the loss of oxygenated functionalities. Notably, TAAC-500 exhibits a peak at 870 cm^−1^, absent in both TABP and TAAC-800, consistent with aromatic C–H out of plane bending.^29^ This observation aligns with the concept “combustion continuum” model, where increasing temperature promoted the condensation of small aromatic structures into larger conjugated sheets.^32^ Together, these results confirm that pyrolysis significantly alters surface chemistry, reducing polar functionalities while promoting of alkene or aromatic ring, whereas these bands were absent in aromatic condensation. Although thermal activation improves carbon aromaticity, the simultaneous loss of surface oxygenated groups can reduce the number of available hydrogen-bonding sites, thereby limiting sorbate interactions compared to the raw biomass.

**Fig. 2:**
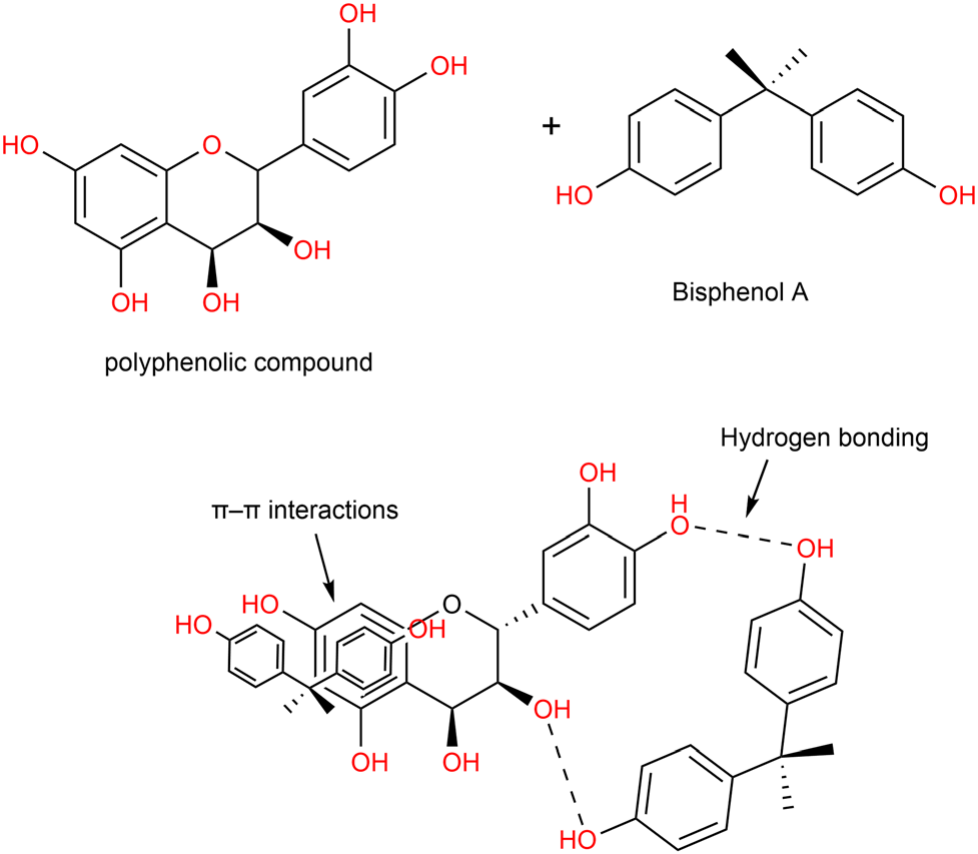
Proposed interaction mechanisms between BPA and polyphenolic surface functionalities of TABP/TAAC, illustrating hydrogen bonding between hydroxyl groups and aromatic π–π stacking with graphitic domains formed during activation.

FT-IR spectra recorded after BPA adsorption (Fig. 1C and D) provided evidence of interaction mechanisms. The enhanced intensity of the O–H stretching band around 3300 cm^−1^ and emergence of a new band at around 1160 cm^−1^ indicate hydrogen bonding between BPA hydroxyl groups and surface functionalities.^33^ In addition, a shift in aromatic C=C stretching vibration around 1600 cm^−1^ in BPA-bound TABP suggests π–π electron interactions between aromatic moieties of BPA and sorbent surfaces.^17^ Shifts observed in O–H and aromatic C=C bands upon BPA adsorption further confirm hydrogen bonding and π–π interactions. These findings support deriving the mechanism of BPA adsorption onto TABP and TAAC, which involves hydrogen bonding and π–π interactions as dominant interaction models (Fig. 2). The FTIR spectra of TAAC-800 exhibit a noticeable reduction of oxygen-containing functional group bands relative to TAAC-500 (Fig. 1A (inset)), suggesting thermal degradation of surface functionalities at higher activation temperatures. The near-complete absence of oxygenated group bands (broad O–H stretching at ∼3340 cm^−1^ and C–O stretching at ∼1031 cm^−1^) coupled with dominant aromatic C=C vibrations indicates excessive loss of polar functional groups and advanced graphitization. These structural changes eliminate key hydrogen-bonding sites and reduce chemical affinity for BPA, directly explaining the markedly poorer adsorption performance of TAAC-800. Further quantitative insights into surface chemical states could be obtained through advanced surface characterization techniques such as XPS analysis, however, this was beyond the scope of the present work and will be considered in future studies to further clarify the effects of high-temperature activation on surface chemistry.

Thermogravimetric analysis (TGA) revealed further structural differences between TABP and TAAC-500 (Fig. 1B). In both materials, an initial ∼13% weight loss was observed, attributed to moisture evaporation. For TABP, subsequent 35% and 28% weight losses corresponded to the decomposition of hemicellulose/cellulose and lignin, respectively, consistent with their known thermal stabilities.^24^ In TAAC-500, a single major weight loss of ∼46% was observed, reflecting the concurrent decomposition of residual hemicellulose, cellulose, and lignin. The reduced overall weight loss in TAAC-500 confirms that surface activation effectively removes thermally unstable biomass components.

**Fig. 3:**
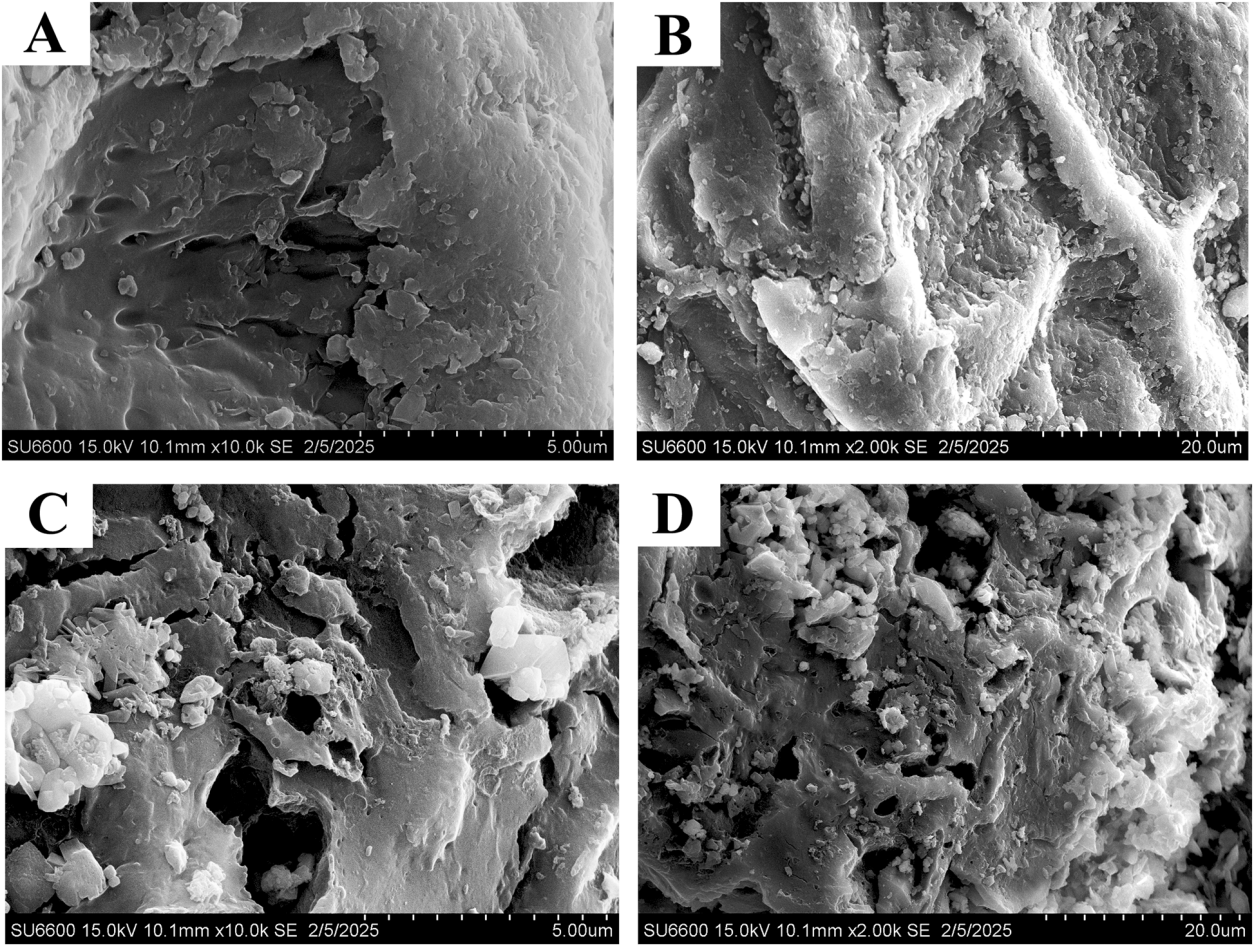
SEM characterization of TABP and TAAC-500. (A and B) SEM images of TABP showing irregular surface morphology; (C and D) SEM images of TAAC-500 revealing increased porosity after pyrolysis.

**Fig. 4:**
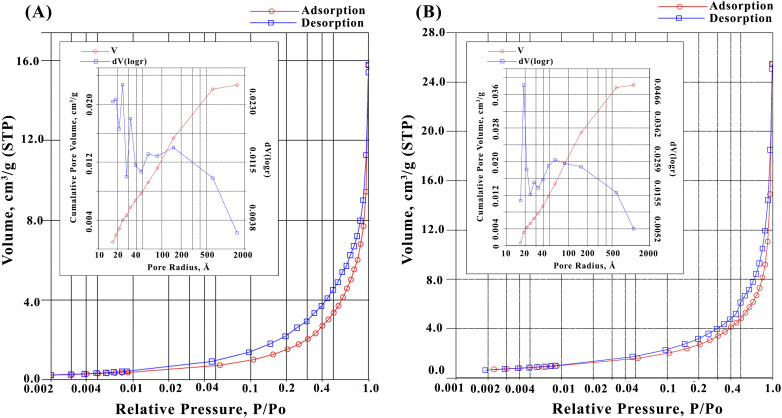
BET nitrogen adsorption–desorption isotherms and pore-size distribution (insets) of (A) TABP and (B) TAAC-500.

SEM-EDX and BET analysis further provided insights into the morphological and compositional modifications. SEM micrographs and EDX analysis confirm morphological and compositional transformation of TABP upon pyrolysis at 500 °C. The raw TABP (Fig. 3 A and B) shows relatively smooth, laminated surfaces with limited visible porosity, whereas the activated carbon TAAC-500 (Fig. 3C and D) exhibits extensive pore development, fractured surfaces, and interconnected cavities formed during carbonization. This indicates substantially higher pore area coverage in activated samples and a predominance of small pore features with a small number of larger cavities, a morphology that favors both increased surface area and enhanced mass-transport pathways. EDX analysis revealed compositional shifts with TABP containing carbon (37.9%), oxygen (53.1%), and calcium (8.2%), whereas TAAC-500 showed increased carbon (53.4%) and calcium (14.3%), with reduced oxygen (32.3%), reflecting enhanced carbonization and mineral retention after activation, consistent with devolatilization and concentration of inorganic ash. The BET analysis (Fig. 4) confirmed an increase in the specific area of TABP to TAAC-500 from 8.00 m^2^ g^−1^ to 9.48 m^2^ g^−1^, and average pore size from 6.54 nm to 7.04 nm. However, the relatively low surface area of TAAC-500 compared to other activated carbons can be attributed to limited pore evolution under the selected activation conditions, where partial KOH–biomass reactions at moderated pyrolysis temperatures led to restricted micro/mesopore development. In contrast to many high-performance activated carbons, the adsorption behaviour observed in the present study suggests a functionality-dominated mechanism, where specific surface chemical interactions play a more critical role than total surface area. Despite the low measured surface area, the presence of aromatic domains and residual functional groups still facilitated efficient BPA adsorption through π–π interactions and hydrogen bonding. It is worth noting that BPA adsorption is not solely governed by total surface area, instead, accessibility and chemical affinity of active sites often play a more dominant role. As seen in TAAC-800, increasing activation temperature does not necessarily improve adsorption performance, as excessively high thermal treatment can collapse pores and remove oxygenated functional groups responsible for strong adsorbate interactions, resulting in poor adsorption performances.

Although chemical activation modifies both the textural and chemical characteristics of the carbon surface, the adsorption behaviour observed in this study indicates that BPA uptake is not solely governed by surface area enhancement. Although TAAC-500 exhibits only a marginal increase in BET surface area (from 8.00 m^2^ g^−1^ for TABP to 9.48 m^2^ g^−1^), TAAC-500 exhibited altered adsorption behaviour compared to the raw biochar, reflecting changes in surface chemistry rather than pore abundance. Pyrolysis at 500 °C promotes aromatic condensation, as evidenced by the emergence of a distinct aromatic C–H out-of-plane bending peak at 870 cm^−1^ and strengthened C=C skeletal vibrations. These structural changes enhance π–π electron–donor–acceptor interactions with BPA's aromatic rings, leading to stronger binding affinity (higher *K*_L_ = 0.1113 L mg^−1^, lower *R*_L_ = 0.018 according to the Langmuir isotherm model) and faster uptake (>95% removal in 30 min) compared to raw TABP. However, the simultaneous reduction in oxygenated functional groups, as evidenced by diminished O–H and C–O bands, decreases the total number of hydrogen bonding sites, resulting in a lower overall adsorption capacity (*q*_max_ = 7.10 mg g^−1^*vs.* 36.10 mg g^−1^ for TABP). This trade-off highlights that mild activation improves kinetics and affinity through excessive modification or loss of specific surface functionalities, but sacrifices capacity due to partial loss of polar functionalities, outweighing the benefits gained from limited pore development, emphasizing the sensitivity of BPA adsorption to functional group composition rather than total surface area.

## Adsorption performance–raw material

### Effect of adsorbent dosage on BPA removal

**Fig. 5:**
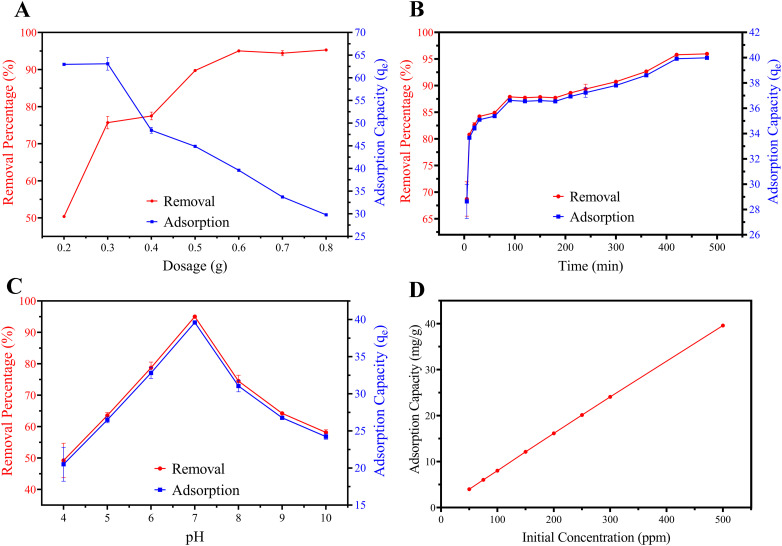
Adsorption performance of TABP: (A) effect of dosage, (B) effect of contact time, (C) effect of pH, (D) effect of initial BPA concentration on adsorption. Results confirm optimal adsorption performance at a 0.6 g dosage level at pH 7 in 480 min. Error bars represent mean ± standard deviation from triplicate experiments (*n* = 3), and in certain scenarios, it may be smaller than the symbol size.

Investigating the effect of adsorbent dosage is a critical step for maximizing contaminant removal. The bark powder dosage ranging from 0.2 g to 0.8 g (4 to 16 g L^−1^) was evaluated at room temperature and pH 7 with an initial BPA concentration of 500 mg L^−1^. The relationship between the dosage, adsorption capacities, and removal percentage of BPA is illustrated in Fig. 5. As shown in Fig. 5A, the BPA removal efficiency increased with increasing adsorbent dosage. At 0.2 g, the removal percentage was 50.38 ± 0.16%, while at 0.6 g sorbent concentration, the removal efficiency significantly increased to 95.04 ± 0.14%. Beyond this dosage, the removal rate plateaued, indicating that additional adsorbents are no longer contributing to BPA uptake due to the limited availability of free solute molecules. At this point, solute becomes the limiting factor, and the unsaturated active sites on the sorbent compete with the BPA molecules. Thus, 0.6 g was identified as the optimal dosage for subsequent studies.

In contrast, adsorption capacities (mg g^−1^, *q*_*e*_) representing the solid-phase equilibrium concentration of BPA,^29^ were inversely related to dosage, with the highest adsorption capacity of 62.98 ± 0.20 mg g^−1^, which was observed at the lowest sorbent dosage of 0.2 g. This reflects greater site saturation at lower dosages, as reported in other biosorbent studies.^34^

Further, these findings highlight the importance of balancing adsorbent dosage to adsorption capacity when designing efficient bio-based adsorption systems for BPA removal in water treatment. Preliminary desorption trials indicate regeneration is feasible, warranting further investigation for cyclic operation.

### Effect of contact time on adsorption

Kinetic studies were performed using a TABP dosage of 0.6 g at pH 7 and room temperature, with the BPA concentration maintained at 500 mg L^−1^ and the contact times ranging from 5 to 480 minutes. As shown in Fig. 5B, BPA removal was rapid during the initial stages, with ∼70% uptake achieved within the first five minutes. The equilibrium was reached after ∼480 minutes (4 hours).

The fast initial uptake is attributed to the abundant, readily accessible surface-bound active sites on the sorbent. The slower, subsequent adsorption phase reflects long-range interparticle diffusion and progressive inner-pore filling. Such biphasic behaviour has been reported in other biomass-derived adsorbents.^21^ These findings demonstrate the suitability of TABP for applications requiring rapid BPA contaminant reduction.

### Effect of pH on BPA adsorption

Solution pH strongly influences both the ionization of BPA and the surface charge of the adsorbent.^18^ Adsorption experiments were conducted across a pH 4–10 with 0.6 g of TABP and 500 mg L^−1^ BPA. Removal efficiencies and adsorption capacities are illustrated in Fig. 5C. Accordingly, the BPA removal increased gradually from pH 4 to 7, achieving a maximum efficiency of 95.04 ± 0.14% (*q*_*e*_ = 39.60 ± 0.06 mg g^−1^) at neutral conditions. Beyond pH 7, removal declined, consistent with findings for other lignocellulosic adsorbents.^35^

### Effect of initial BPA concentration on adsorption

The effect of initial BPA concentration on adsorption was studied with a 0.6 g adsorbent dosage at pH 7 for a 24 h equilibrium time, using BPA solutions ranging from 50 mg L^−1^ to 500 mg L^−1^. Fig. 5D shows that the adsorption capacity increased with the initial BPA concentration, from 4.00 to 39.60 mg g^−1^, which can be explained by mass transfer effect. Higher BPA concentrations in the solution increase the driving force across the solution–adsorbent interface to overcome the resistance at the mass transfer boundary.^36^ Importantly, TABP maintained effective adsorption performance across a wide concentration range, demonstrating its robustness for treating contaminated water.

### Point zero charge (pH_pzc_) of adsorbent

**Fig. 6:**
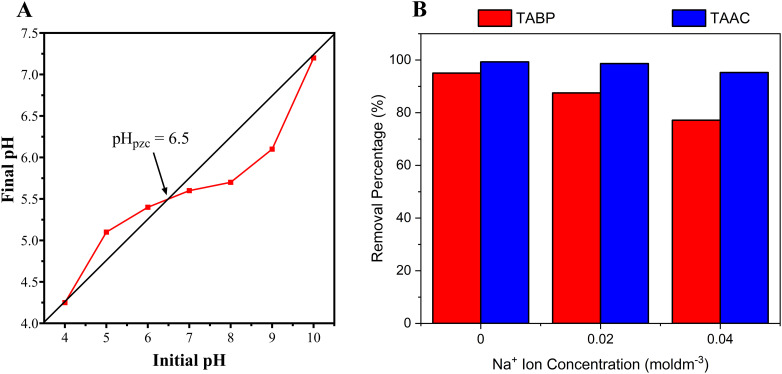
(A) Point of zero charge (pH_pzc_) of TABP (∼6.5), indicating that BPA uptake is favoured near neutral pH due to minimal electrostatic repulsion. (B) Effect of ionic strength on adsorption, indicating a stronger influence of ionic strength for TABP, whereas TAAC 500 is less affected by electrolyte concentration.

Given the dependence of BPA removal with pH on the adsorbent surface, the point zero charge (pH_pzc_) was determined with respect to the correlation between the ‘surface charge density’ and ‘charge on BPA molecules’. The pH_pzc_ of TABP was determined to be ∼6.5 (Fig. 6A). The pH_pzc_ value indicates that the adsorbent surface is deprotonated, bearing negative surface charges at pH values greater than pH_pzc_, which favours the adsorption of cationic species. Conversely, at pH values lower than the pH_pzc_, the surface is positively charged, promoting the adsorption of anionic species. At pH <PH_pzc_, in acidic conditions, the adsorbent surface is positively charged, leading to competition between H^+^ ions and BPA for the adsorbent's active sites.^35^ Near pH 7, close to the pH_pzc_ value and neutrality, the interactions are maximized. Under these conditions, the adsorbent surface charge density is neutral, thereby achieving the highest removal capacity.^20^ At alkaline pH > p*K*_a_ of BPA (9.6–10.2), both BPA and TABP surfaces become negatively charged as BPA exists in its deprotonated forms (BPA^2−^ and HBPA^−^) and the adsorbent surface bears negative charge (being higher than the pH_pzc_), resulting in electrostatic repulsion and reduced adsorption.^20^ This behaviour highlights the dominant role of electrostatic interactions in governing BPA removal.

### Effect of ionic strength on adsorption

The influence of ionic strength was evaluated using Na^+^ ion concentrations of 0.02 and 0.04 M in BPA solutions (Fig. 6B). Removal efficiencies decreased from 95.0% to 87.50% and 77.16% in 0.02 and 0.04 M Na^+^ ion concentrations, respectively. This reduction is attributed to competition between Na^+^ ions and BPA molecules for the available active sites. The results reflect the complexity of adsorption in industrial effluents, where competing ions may reduce the sorbent efficiency. The stronger influence of ionic strength observed for TABP is consistent with its pH_pzc_ value (∼6.5), indicating a greater contribution of electrostatic interactions to BPA adsorption near neutral pH. Electrostatic screening by Na^+^ ions, therefore, reduces adsorption efficiency. In contrast, adsorption on TAAC-500 appears to rely more strongly on specific surface interactions such as π–π interactions and hydrogen bonding, which are less affected by electrolyte concentration, resulting in lower Na^+^ sensitivity. While pH_pzc_ measurements provided valuable insight into surface charge behaviour, further characterization techniques, such as zeta potential measurements, would enable a more quantitative assessment of the adsorption mechanisms. These analyses are therefore suggested for future studies to complement the present findings.

## Adsorption performance–activated carbons

The adsorption behaviour of activated carbon prepared at 500 °C (TAAC-500) was evaluated to compare it with that of the raw material. Although TAAC-500 was prepared *via* chemical activation using KOH, its adsorption behaviour in the present system is better described as that of a surface functionality-dominated carbonaceous adsorbent rather than by extensive pore development, which explains its deviation from the high surface-area-performance correlations commonly reported for conventional activated carbons.

**Fig. 7:**
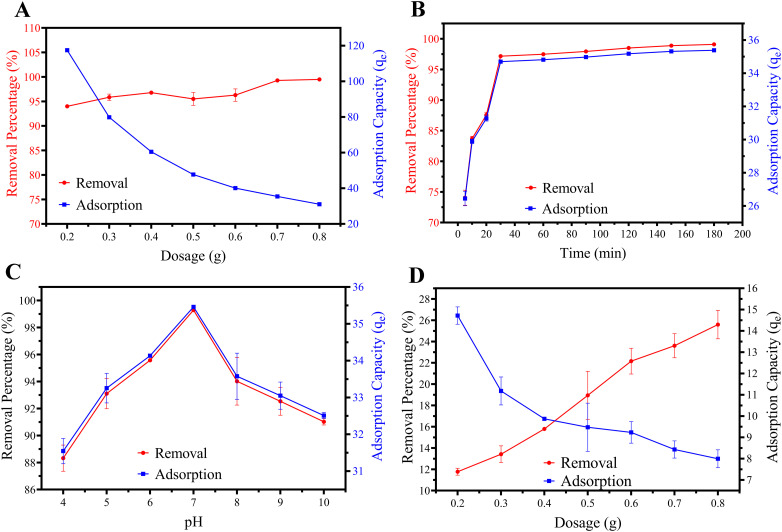
Adsorption performance of KOH-modified carbonaceous adsorbent: (A) effect of dosage of TAAC-500, (B) effect of contact time of TAAC-500, (C) effect of pH of TAAC-500, (D) effect of dosage of TAAC-800. TAAC-500 demonstrates faster equilibrium and higher removal efficiency at low dosage. TAAC-800 yielded poor performance, where the highest removal efficiency was much lower than both TABP and TAAC-500. Error bars represent mean ± standard deviation from triplicate experiments (*n* = 3), and in certain scenarios, they may be smaller than the symbol size.

As shown in Fig. 7A, TAAC-500 demonstrated a higher removal efficiency at lower dosages. At a dosage of 0.2 g, the removal reached 94.01 ± 0.11%, whereas the TABP removed only 50.38 ± 0.16% at the same dosage level. At the optimum dose of 0.7 g (14 g L^−1^), the maximum removal was 99.29 ± 0.00% with an adsorption capacity of 35.46 mg g^−1^. These results confirm the efficiency of activation in enhancing removal performance, while optimizing dosage reduced material requirements. The effect of contact time on adsorption by TAAC-500 is illustrated in Fig. 7B. Unlike TABP, where equilibrium required 480 min, TAAC-500 achieved >95% removal within 30 min. This rapid uptake indicates the accessibility of adsorption sites and efficient pore diffusion after activation. Similar rapid kinetics have been reported for other biomass-derived adsorbents, such as palm-kernel-shell biochar (93% removal within 60 min) and potato-peel activated carbon (∼70 min).^11^ This enhanced kinetics and affinity of TAAC-500 despite minimal pore development highlights the dominant role of surface chemistry over physical porosity. Increased aromatic domains facilitate specific surface interactions, including possible π–π interactions, while residual oxygenated groups support hydrogen bonding, collectively enabling rapid and favourable BPA binding.

The pH influence on adsorption showed the same trend as TABP, with optimal adsorption at pH 7 (Fig. 7C). Interestingly, ionic strength had less influence on TAAC-500 compared to TABP.

By contrast, activation at 800 °C (TAAC-800) yielded poor performance (Fig. 7D). The highest removal efficiency was only 25.60 ± 1.34% at 0.8 g dosage, much lower than both TABP and TAAC-500 at equivalent or lower dosages. Consequently, TAAC-800 was not further investigated. The decline in performance is attributed to the loss of oxygenated surface functional groups and excessive graphitization at high temperature, reducing active adsorption sites.

Although TAAC-500 exhibits a relatively low BET surface area (9.48 m^2^ g^−1^), which contrasts with typical activated carbons (>500 m^2^ g^−1^), it demonstrates high BPA removal efficiency (>95%) under the studied conditions. It should be noted that high BPA removal efficiency at the tested concentrations does not necessarily correspond to high gravimetric adsorption capacity, particularly under conditions where adsorption is governed by site-specific interactions rather than extensive pore filling. This highlights that BPA adsorption in this system is not governed primarily by total surface area, but rather by surface chemical functionality and site-specific interactions. BPA is a hydrophobic aromatic molecule containing phenolic groups, and its adsorption has been reported to depend strongly on π–π electron donor–acceptor interactions, hydrogen bonding, and electrostatic attractions with oxygen-containing surface groups rather than solely on pore volume or surface area. In such cases, accessible and chemically active adsorption sites can outweigh the contribution of extensive but poorly interactive surface area. *Terminalia arjuna* bark's natural polyphenols and tannins provide abundant sites for π–π stacking and hydrogen bonding, outperforming porosity-limited mechanisms in some high-surface-area materials. The KOH activation at 500 °C introduces surface defects and oxygen-functional groups that enhance chemical affinity toward BPA, while excessive activation severity may compromise functional group density or accessibility. Therefore, the adsorption behaviour observed for TAAC-500 highlights that pore accessibility and surface chemistry are more critical than absolute BET surface area for BPA uptake, particularly for aromatic endocrine-disrupting compounds.

## Adsorption isotherms

Adsorption isotherms provide critical insights into the interaction between the adsorbate and the adsorbent surface under the equilibrium conditions. Experiments were performed with 0.6 g of adsorbent at pH 7 and room temperature, using BPA concentrations ranging from 50 to 500 mg L^−1^ and an equilibrium time of 24 hours. To evaluate the adsorption behaviour, equilibrium data were analysed using multiple isotherm models, including Langmuir, Freundlich, Hill, Temkin, and Jovanovich. The fitted parameters and regression coefficients (*R*^2^) of each model are summarized in Table 2. These models provide complementary insights into surface properties, adsorption mechanisms, and interaction energetics. These models were applied to assess adsorption affinity, surface heterogeneity, and possible cooperative effects. The mechanistic interpretations were derived by integrating trends across multiple isotherm models with material characterization results, rather than relying on a single model. This approach allows a more balanced and physically realistic interpretation of BPA adsorption behaviour on chemically modified biomass-derived carbonaceous adsorbents.

**Table 2:** Comparison of adsorption isotherm model parameters for BPA adsorption onto TABP and TAAC-500. Values illustrate differences in adsorption capacity, surface heterogeneity, and binding cooperativity across Langmuir, Freundlich, Temkin, Hill, and Jovanovich models

Model	Parameter	TABP	TAAC-500	Interpretation
Langmuir	*q*_max_ (mg g^−1^)	36.101	7.101	Higher monolayer capacity in TABP–affinity increased after activation
	*K*_L_ (mg g^−1^)	0.01425	0.1113	Affinity constant–higher for TAAC.
	*R*_L_	0.7388	0.0184	Favourable adsorption in both–stronger in TAAC.
	*R* ^2^	0.965	0.8345	Better fit for TABP
Freundlich	1/n	0.9181	1.54146	Favourable adsorption (TABP)–cooperative adsorption (TAAC)
	*K*_F_ (L mg^−1^)	2.569	1.435	Higher adsorption capacity for TABP.
	*R* ^2^	0.9568	0.9144	Good fit for both
Temkin	*b*	174.152	121.667	Stronger adsorbate–adsorbent interaction in TABP.
	*A*_T_	0.9408	0.5325	Higher binding constant for TABP.
	*R* ^2^	0.97947	08 776	Good fit for TABP
Hill	*K*_D_	26.800	35.232	Dissociation constant lower for TABP
	*n*_H_	1.577	2.1665	Positive cooperativity in both; stronger for TAAC.
	*R* ^2^	0.9969	0.92481	Good fit for TABP
Jovanovich	*K*_J_ (mg g^−1^)	0.09527	0.17314	Higher energy parameter in TAAC.
	*R* ^2^	0.9960	0.96203	Good fit for both

The Langmuir model was employed to estimate the seeming monolayer adsorption capacity and relative binding affinity, assuming a finite number of energetically equivalent adsorption sites. Although the strict assumptions of surface homogeneity and identical adsorption energies may not be fully applicable to biomass-derived carbonaceous materials, the model remains useful for comparative evaluation of adsorption strength. The dimensionless separation factor (*R*_L_) was used to assess adsorption favourability over the investigated concentration range.^7,23^

The Jovanovich isotherm, which also describes localized monolayer adsorption without lateral interactions, but includes a probabilistic interpretation of adsorption-desorption processes, was applied to further examine adsorption behaviour, particularly at higher equilibrium concentrations. Differences between Langmuir and Jovanovich fittings provide insight into deviations from idealized monolayer saturation behaviour.^37,38^

In contrast, the Freundlich isotherm describes adsorption on energetically heterogeneous surfaces, allowing assessment of surface heterogeneity and adsorption intensity. The Freundlich heterogeneity parameter (1/n) was used to qualitatively evaluate adsorption favourability and the potential contribution of site-energy distributions rather than to infer multilayer adsorption in a strict physical interpretation.^17^

The Hill isotherm model was employed to examine potential cooperative effects during BPA adsorption. The Hill coefficient was used as a phenomenological parameter to indicate whether adsorption events may influence subsequent BPA binding on the adsorbent surface. In this context, values of the Hill coefficient *n*_H_ > 1 suggest apparent positive cooperativity, whereas values of *n*_H_ = 1 indicate non-cooperative adsorption behaviour. It is emphasized that this interpretation reflects collective adsorption trends rather than direct molecular-level interactions.^37,39–42^

**Fig. 8:**
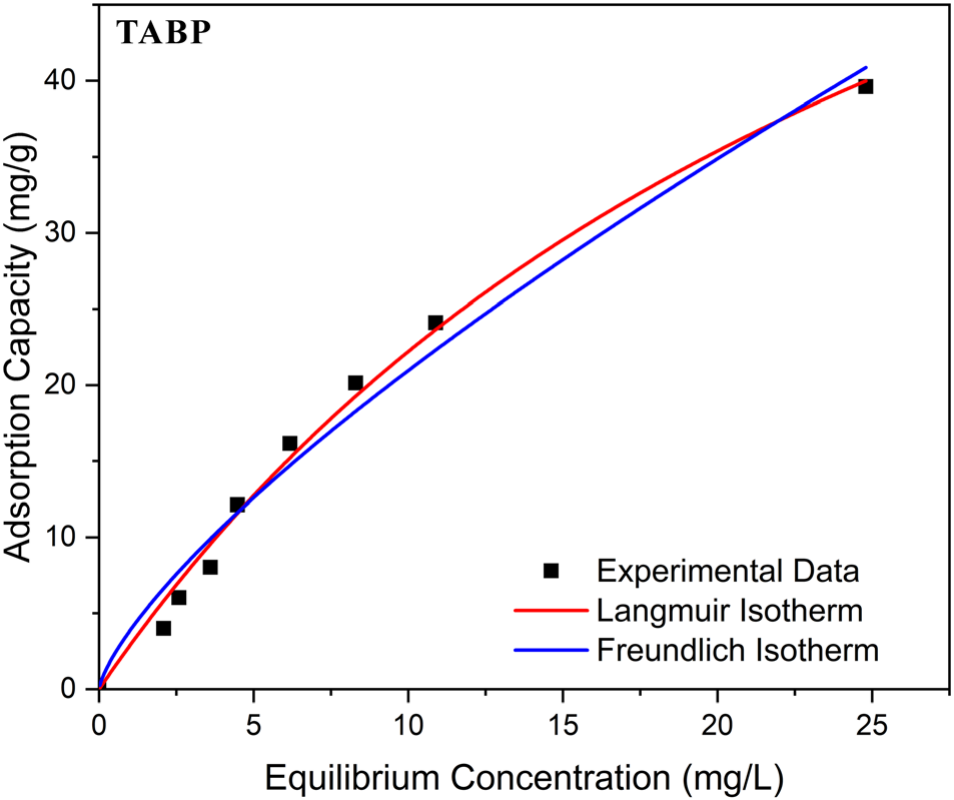
Langmuir and Freundlich isotherm model fits for BPA adsorption onto TABP at pH 7 and room temperature. Symbols represent experimental equilibrium data, while solid lines correspond to model fits. The comparison illustrates the relative applicability of monolayer (Langmuir) and heterogeneous multilayer (Freundlich) adsorption descriptions.

The Temkin isotherm assesses changes in adsorption energy as a function of surface coverage, accounting for indirect adsorbate–adsorbate interactions. This model provides complementary information on adsorption energetics without assuming a constant heat of adsorption.^43,44^ To facilitate independent evaluation of model fitting quality, experimental data points and corresponding fitted curves are explicitly presented for both isotherm and kinetic analyses (Fig. 8).

The Langmuir model provided a good fit for TABP (*R*^2^ = 0.965), indicating monolayer adsorption on homogeneous active sites with a maximum capacity (*q*_max_) of 36.10 mg g^−1^ (Fig. 8). The *R*_L_ value (0.739) suggested favourable adsorption. In contrast, TAAC-500 showed a much lower *q*_max_ (7.10 mg g^−1^) and weaker fit (*R*^2^ = 0.835), though the *R*_L_ value of 0.018 confirmed highly favourable adsorption at higher affinity (*K*_L_ = 0.1113). The lower adsorption capacity of TAAC-500 compared to TABP can be attributed to changes in surface chemical functionality induced by chemical activation. While KOH activation introduces structural defects and enhances aromaticity, it can simultaneously reduce the abundance or accessibility of polar oxygen-containing functional groups that facilitate BPA binding. Although TAAC-500 exhibits stronger site-specific interactions, the overall number of effective adsorption sites may be reduced relative to the TABP.

The Freundlich model also described the data reasonably well (*R*^2^ = 0.957 for TABP and 0.914 for TAAC-500). For TABP, 1/n = 0.918 (<1) confirmed favourable adsorption on heterogeneous sites (Fig. 8). This observation is consistent with the presence of multiple surface functional groups identified by FTIR analysis, which can contribute to a distribution of adsorption site energies. For TAAC-500, 1/n = 1.54 (>1) suggested cooperative adsorption, consistent with increased affinity but reduced available sites after activation. The Freundlich behaviour suggests that surface chemistry, rather than total surface area alone, plays a dominant role in BPA uptake.

**Fig. 9:**
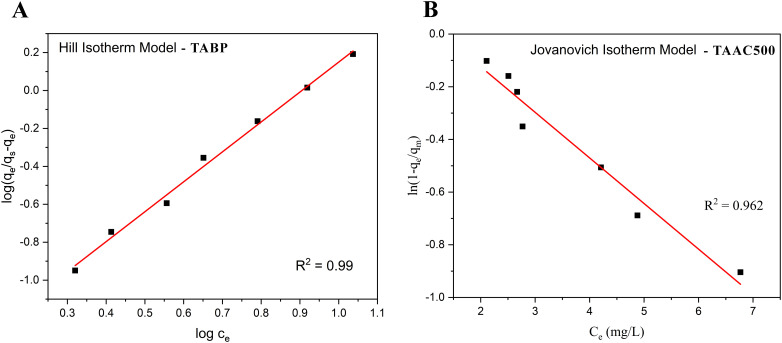
Best-fitting adsorption isotherm models for BPA adsorption onto TABP and TAAC-500 under identical experimental conditions. Symbols denote experimental data, and lines represent model predictions. The plots highlight the adsorption behaviour and fitting quality of the raw biomass and KOH-modified carbonaceous adsorbent.

The Hill isotherm revealed apparent positive cooperativity for both adsorbents, with Hill coefficients *n*_H_ of 1.58 (TABP) and 2.17 (TAAC). The high correlation for TABP (*R*^2^ = 0.997) indicated that this behaviour may arise from local surface environments where initial BPA adsorption facilitates subsequent adsorption through enhanced π–π interactions or hydrogen bonding at neighbouring sites (Fig. 9A). The positive cooperativity (*n*_H_ > 1) indicates interdependent adsorption events following initial BPA binding, without implying an increase in the total number of adsorption sites. This interpretation reflects an overall adsorption trend rather than direct molecular-level cooperative binding.

The Jovanovich model also fits well (*R*^2^ = 0.996 for TABP; 0.962 for TAAC), supporting localized adsorption behaviour, showing gradual saturation trends consistent with non-ideal monolayer adsorption (Fig. 9B). The Temkin model provided a good fit for TABP (*R*^2^ = 0.979) with higher Temkin constant *b* (174.15 J mol^−1^) for TABP reflecting gradual decrease in adsorption energy with increasing surface coverage, suggesting that indirect adsorbate–adsorbate interactions contribute to the adsorption process.,

Taken together, the higher Hill coefficient (*n*_H_ = 2.17 *vs.* 1.58 for TABP) and Freundlich 1/n > 1 for TAAC-500 indicate apparent positive cooperativity, consistent with enhanced π–π interactions on more condensed aromatic surfaces formed during mild pyrolysis. Both parameters suggest that adsorption at one site increases the affinity of neighbouring sites, rather than implying classical multilayer adsorption on a highly heterogeneous surface. This cooperative effect is attributed to extended π–π interactions across condensed aromatic domains, particularly enhanced in TAAC-500. This supports the overall conclusion of favourable site-limited adsorption with cooperative effects, consistent with dominant specific interactions rather than purely surface-area-driven physisorption. However, this behaviour should be interpreted as evidence of interdependent adsorption events rather than enhanced adsorption capacity. In the present system, cooperative effects likely arise from localized surface rearrangements or adsorbate–adsorbate interactions following initial BPA binding. While such cooperativity enhances adsorption affinity, it does not compensate for the reduced density of adsorption-active functional groups, explaining the lower equilibrium uptake observed for TAAC-500. Despite the relatively low BET surface area of TAAC-500, its high BPA removal efficiency underscores the importance of pore accessibility and surface functional chemistry over total surface area alone. The reduced adsorption performance of TAAC-800 is likely associated with the partial loss of oxygen-containing functional groups and increased structural ordering induced by high-temperature activation. It is important to note that the adsorption isotherm models employed in this study are used as phenomenological tools to describe equilibrium behaviour rather than as definitive proof of a single adsorption mechanism.

## Adsorption kinetics

**Fig. 10:**
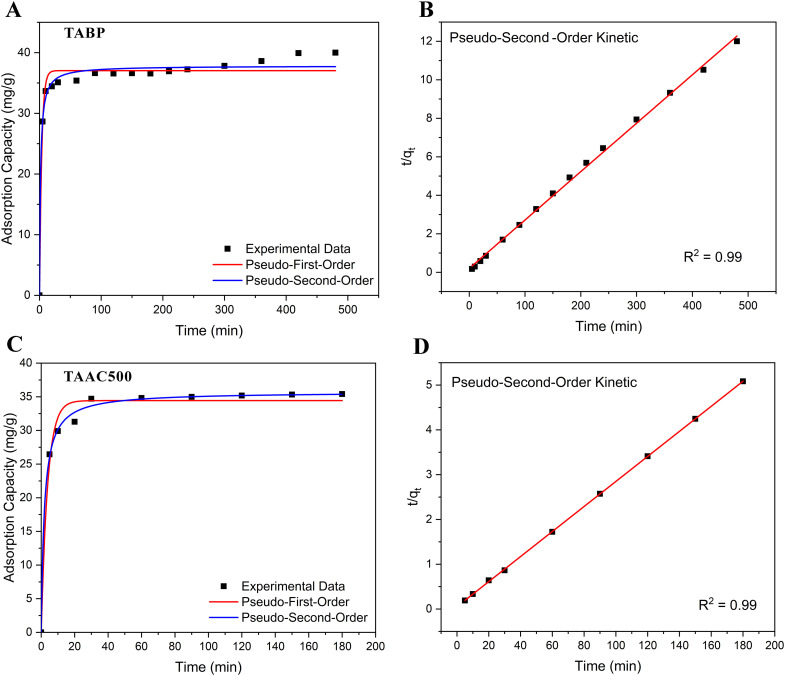
Pseudo-first-order (PFO) and pseudo-second-order (PSO) kinetic model fits for BPA adsorption onto (A) TABP and (C) TAAC-500. Experimental data points are shown as symbols, while fitted curves represent kinetic model predictions. The linear forms of pseudo-second-order kinetic plots of (B) TABP and (D) TAAC-500 demonstrate the superior agreement of the PSO model with experimental data for both adsorbents.

In order to determine the mechanism and rate-controlling steps in the process, the adsorption kinetics were evaluated using pseudo-first-order (PFO) and pseudo-second-order (PSO) models (Fig. 10). The kinetic parameters are presented in Table 3.

**Table 3:** Kinetic parameters for BPA adsorption onto TABP and TAAC-500 derived from pseudo-first-order (PFO) and pseudo-second-order (PSO) models. Results indicate superior fit to the PSO model for both materials, consistent with chemisorption-dominated mechanisms

Model	Parameter	TABP	TAAC-500
Pseudo-first-order (PFO)	*K*_1_ (min^−1^)	0.0073	0.0291
	Theoretical *q*_*e*_ (mg g^−1^)	8.856	5.433
	Experimental *q*_*e*_ (mg g^−1^)	39.22	35.40
	*R* ^2^	0.671	0.882
Pseudo-second-order (PSO)	*K*_2_ (g mg^−1^ min)	0.003	0.0147
	Theoretical *q*_*e*_ (mg g^−1^)	39.777	35.765
	Experimental *q*_*e*_ (mg g^−1^)	39.22	35.40
	*R* ^2^	0.998	0.999

The PFO model showed moderate correlation for both TABP (*R*^2^ = 0.671) and TAAC (*R*^2^ = 0.882). Furthermore, the theoretical *q*_*e*_ values (8.86 mg g^−1^ for TABP and 5.43 mg g^−1^ for TAAC) deviated significantly from the experimental *q*_*e*_ values (39.22 mg g^−1^ and 35.40 mg g^−1^, respectively), indicating that PFO does not adequately describe the adsorption process.

In contrast, the PSO provided excellent fits (*R*^2^ = 0.998 for TABP and 0.999 for TAAC), with theoretical *q*_*e*_ values of 39.78 mg g^−1^ TABP and 35.77 mg g^−1^ TAAC, which are comparable with the experimental values of 39.22 and 35.40, respectively. This strongly suggests that the rate-limiting step is chemisorption, involving valence forces through electron sharing or exchange between BPA molecules and the adsorbent surface.^15^ Further, the *q*_*e*_ values reported here (39.2 mg g^−1^) are comparable to or surpass several reported sorbent materials, including rice husk ash (28–35 mg g^−1^) and durian peel (25–38 mg g^−1^).

The isotherm and kinetic analyses confirm that BPA adsorption onto TABP and TAAC is favourable, localized adsorption behaviour consistent with site-specific interactions under the studied conditions, with positive cooperativity indicating interdependent adsorption events following initial BPA binding. TABP showed higher overall capacity, while TAAC-500 exhibited stronger affinity and faster kinetics.

The low surface areas of TABP and TAAC-500 are significantly lower than those of most of the reported biochars.^49,50^ However, their performance aligns with or exceeds several low-porosity biochar, such as grapefruit peel biochar (1.71 m^2^ g^−1^, effective *via* H-bonding and π–π), *Ulva prolifera* N-doped biochar (25.43 m^2^ g^−1^), and sugarcane bagasse biochar (14.3 m^2^ g^−1^), where adsorption relies predominantly on surface functional groups enabling hydrogen bonding, π–π stacking, and electrostatic interactions rather than physical pore filling (Table 4). *Terminalia arjuna* bark's inherent polyphenols and tannins provide specific chemical affinity for aromatic pollutants like BPA, establishing minimally processed or mildly activated precursors as sustainable, low-energy alternatives particularly suited for resource-limited contexts. Taken together, these results demonstrate that activation-induced changes in surface chemistry can enhance adsorption affinity while simultaneously reducing adsorption capacity, highlighting the importance of balancing functional group preservation and structural modification when designing bio-derived carbon adsorbents for BPA removal.

**Table 4:** Comparison of selected bio-derived adsorbents for BPA removal from aqueous solutions, ordered by increasing BET surface area (SBET) to highlight performance across low-to high-porosity materials

Precursor	Preparation method	SBET (m^2^ g^−1^)	Key highlights	Reference
Grapefruit peel biochar	Pyrolysis at 400 °C	1.71	Extremely low surface area, adsorption *via* H-bonding and π–π EDA interactions	Wang *et al.* (2020)^3^
*Terminalia arjuna* bark	Cleaned and dried	8.00	High capacity from natural polyphenols/tannins, positive cooperativity, chemistry-driven despite low porosity	Current study
*Terminalia arjuna* bark	KOH, 500 °C	9.48	Faster kinetics (>95% in 30 min), higher affinity; low porosity but effective *via* π–π and residual groups	Current study
Sugarcane bagasse biochar	Pyrolysis at 400 °C	14.3	Low surface area but good capacity *via* functional groups, H-bonding, electrostatics	Ponnuchamy *et al.* (2025)^45^
*Ulva prolifera* (marine algae)	N-doped hydrothermal carbonization	25.43	Low surface area for algal biochar; enhanced at high temp/ionic strength; pseudo-second-order kinetics	Lu *et al.* (2017)^19^
Banana peels	Pyrolysis at 600 °C	114.3	Improved over raw; regeneration with EDTA	Din *et al.* (2025)^46^
Sunflower stem	KOH, 500 °C	452	π–π and H-bonding; regeneration with NaOH	Lingamdinne *et al.* (2024)^47^
Wheat straw	ZnCl_2_ + Template strategy	2944	Extremely high porosity; pore filling dominant	Shi *et al.* (2022)^48^

## Conclusion

This study investigated the adsorption performance and mechanistic behaviour of *Terminalia arjuna* bark powder (TABP) and its KOH-modified carbonaceous derivative (TAAC) for the removal of bisphenol A (BPA) from aqueous media. By integrating adsorption experiments with surface characterization and equilibrium modelling, the work provides insight into how surface chemistry, rather than surface area alone, governs BPA uptake on bio-derived carbon materials with limited pore development. Despite exhibiting relatively low BET surface areas, both TABP and TAAC-500 demonstrated high BPA removal efficiencies under optimized conditions. The raw biomass showed a higher maximum adsorption capacity, whereas TAAC-500 exhibited stronger binding affinity and faster adsorption kinetics. This contrast highlights the role of chemical activation in modifying surface functionality and adsorption energetics, even when extensive pore development is not achieved. The poor performance of TAAC-800 further confirms that excessive thermal treatment can diminish adsorption efficiency through loss of surface functional groups and increasing structural ordering. Equilibrium and kinetic analyses indicate that BPA adsorption on these materials cannot be explained by a single idealized mechanism. Langmuir and Jovanovich models describe localized adsorption behaviour under equilibrium conditions, while Freundlich and Hill models capture surface heterogeneity and cooperative adsorption effects, respectively. Importantly, these models are interpreted as phenomenological descriptors rather than direct evidence of specific molecular pathways, with mechanistic insights drawn from their combined evaluation alongside FTIR analysis and pH_pzc_ behaviour.

While the present study was conducted under controlled batch conditions using a single target contaminant, the findings offer valuable guidance for the rational design of bio-derived adsorbents where surface chemical affinity is prioritized over maximization of surface area. Future work should focus on incorporating additional surface-sensitive techniques such as zeta potential or XPS analysis, evaluating performance under dynamic flow conditions, and testing adsorption behaviour in complex water matrices to further assess environmental applicability. In summary, this work demonstrates that plant-based carbonaceous materials can serve as effective and sustainable adsorbents for emerging organic contaminants, provided that surface chemistry and adsorption energetics are carefully tailored rather than relying solely on conventional surface area enhancement strategies.

## Author contributions

Conceptualization, P. W. S. and G. R.; methodology, P. W. S., G. R. and A. S. P.; validation, A. S. P. and K. D. A. D.; formal analysis, A. S. P. and K. D. A. D.; investigation, A. S. P. and K. D. A. D.; resources, P. W. S.; data curation, A. S. P. and K. D. A. D.; writing – original draft preparation, A. S. P. and K. D. A. D.; writing – review and editing, P. W. S. and G. R.; visualization, A. S. P. and K. D. A. D.; supervision, P. W. S.; project administration, P. W. S.; funding acquisition, P. W. S. All authors have read and agreed to the published version of the manuscript.

## Conflicts of interest

The authors declare no conflicts of interest.

## Data Availability

All data supporting the findings of this study are contained within the article.

## References

[cit1] Grumetto L., Montesano D., Seccia S., Albrizio S., Barbato F. (2008). Determination of Bisphenol A and Bisphenol B Residues in Canned Peeled Tomatoes by Reversed-Phase Liquid Chromatography. J. Agric. Food Chem..

[cit2] Corrales J., Kristofco L. A., Baylor Steele W., Yates B. S., Breed C. S., Spencer Williams E., Brooks B. W. (2015). Global Assessment of Bisphenol a in the Environment: Review and Analysis of Its Occurrence and Bioaccumulation. Dose Response.

[cit3] Wang J., Zhang M. (2020). Adsorption Characteristics and Mechanism of Bisphenol a by Magnetic Biochar. Int. J. Environ. Res. Publ. Health.

[cit4] Huo X., Chen D., He Y., Zhu W., Zhou W., Zhang J. (2015). Bisphenol-a and Female Infertility: A Possible Role of Gene-Environment Interactions. Int. J. Environ. Res. Publ. Health.

[cit5] Rochester J. R. (2013). Bisphenol A and Human Health: A Review of the Literature. Reprod. Toxicol..

[cit6] Phouthavong-Murphy J. C., Merrill A. K., Zamule S., Giacherio D., Brown B., Roote C., Das P. (2020). Phytoremediation Potential of Switchgrass (Panicum Virgatum), Two United States Native Varieties, to Remove Bisphenol-A (BPA) from Aqueous Media. Sci. Rep..

[cit7] Katibi K. K., Yunos K. F., Man H. C., Aris A. Z., Nor M. Z. M., Azis R. S. (2021). An Insight into a Sustainable Removal of Bisphenol a from Aqueous Solution by Novel Palm Kernel Shell Magnetically Induced Biochar: Synthesis, Characterization, Kinetic, and Thermodynamic Studies. Polymers.

[cit8] Mpatani F. M., Han R., Aryee A. A., Kani A. N., Li Z., Qu L. (2021). Adsorption Performance of Modified Agricultural Waste Materials for Removal of Emerging Micro-Contaminant Bisphenol A: A Comprehensive Review. Sci. Total Environ..

[cit9] Huang F. C., Lee C. K., Han Y. L., Chao W. C., Chao H. P. (2014). Preparation of Activated Carbon Using Micro-Nano Carbon Spheres through Chemical Activation. J. Taiwan Inst. Chem. Eng..

[cit10] Heidarinejad Z., Dehghani M. H., Heidari M., Javedan G., Ali I., Sillanpää M. (2020). Methods for Preparation and Activation of Activated Carbon: A Review. Environ. Chem. Lett..

[cit11] Lazim Z. M., Hadibarata T., Puteh M. H., Yusop Z. (2015). Adsorption Characteristics of Bisphenol a onto Low-Cost Modified Phyto-Waste Material in Aqueous Solution. Water Air Soil Pollut..

[cit12] Şenol Z. M., Gül Ü. D., Gürkan R. (2020). Bio-Sorption of Bisphenol a by the Dried- and Inactivated-Lichen (Pseudoevernia Furfuracea) Biomass from Aqueous Solutions. J. Environ. Health Sci. Eng..

[cit13] Hayoun B., Bourouina-Bacha S., Pazos M., Sanromán M. A., Benkhennouche-Bouchene H., Deflaoui O., Hamaidi-Maouche N., Bourouina M. (2021). Production of Modified Sunflowers Seed Shells for the Removal of Bisphenol A. RSC Adv..

[cit14] Ahsan M. A., Islam M. T., Hernandez C., Kim H., Lin Y., Curry M. L., Gardea-Torresdey J., Noveron J. C. (2018). Adsorptive Removal of Sulfamethoxazole and Bisphenol A from Contaminated Water Using Functionalized Carbonaceous Material Derived from Tea Leaves. J. Environ. Chem. Eng..

[cit15] Lazim Z. M., Hadibarata T., Puteh M. H., Yusop Z., Wirasnita R., Nor N. M. (2015). Utilization of Durian Peel as Potential Adsorbent for Bisphenol A Removal in Aquoeus Solution. J. Teknol..

[cit16] Lazim Z. M., Hadibarata T., Yusop Z., Nazifa T. H., Abdullah N. H., Nuid M., Salim N. A. A., Zainuddin N. A., Ahmad N. (2021). Bisphenol a Removal by Adsorption Using Waste Biomass: Isotherm and Kinetic Studies. Biointerface Res. Appl. Chem..

[cit17] Arampatzidou A. C., Deliyanni E. A. (2016). Comparison of Activation Media and Pyrolysis Temperature for Activated Carbons Development by Pyrolysis of Potato Peels for Effective Adsorption of Endocrine Disruptor Bisphenol-A. J. Colloid Interface Sci..

[cit18] Soni H., Padmaja P. (2014). Palm Shell Based Activated Carbon for Removal of Bisphenol A: An Equilibrium, Kinetic and Thermodynamic Study. J. Porous Mater..

[cit19] Lu J., Zhang C., Wu J., Luo Y. (2017). Adsorptive Removal of Bisphenol A Using N-Doped Biochar Made of Ulva Prolifera. Water Air Soil Pollut..

[cit20] Alves A. C. F., Antero R. V. P., de Oliveira S. B., Ojala S. A., Scalize P. S. (2019). Activated Carbon Produced from Waste Coffee Grounds for an Effective Removal of Bisphenol-A in Aqueous Medium. Environ. Sci. Pollut. Res..

[cit21] Sudhakar P., Mall I. D., Srivastava V. C. (2016). Adsorptive Removal of Bisphenol-A by Rice Husk Ash and Granular Activated Carbon—A Comparative Study. Desalination Water Treat..

[cit22] Amalraj A., Gopi S. (2017). Medicinal Properties of Terminalia Arjuna (Roxb.) Wight & Arn.: A Review. J. Tradit. Complement. Med..

[cit23] Swain K. K., Mishra P. M., Devi A. P. (2018). Biosorption of Praseodymium (III) Using Terminalia Arjuna Bark Powder in Batch Systems: Isotherm and Kinetic Studies. Water Sci. Technol..

[cit24] Shakoor S., Nasar A. (2018). Adsorptive Decontamination of Synthetic Wastewater Containing Crystal Violet Dye by Employing Terminalia Arjuna Sawdust Waste. Groundw. Sustain. Dev..

[cit25] Rao R. A. K., Khatoon A., Ashfaq A. (2016). Application of Terminalia Arjuna as Potential Adsorbent for the Removal of Pb(II) from Aqueous Solution: Thermodynamics, Kinetics and Process Design. Desalination Water Treat..

[cit26] Papodu K., Rao Y. H., Ravindhranath K. (2014). Removal of Zinc from Waste Waters Using New Biosorbents Derived from *Terminalia arjuna*, *Atlantia monophylla* (L.) Correa and *Madhuca indica* Plants. Der Pharma Chemica.

[cit27] Mohanty K., Jha M., Meikap B. C., Biswas M. N. (2005). Preparation and Characterization of Activated Carbons from Terminalia Arjuna Nut with Zinc Chloride Activation for the Removal of Phenol from Wastewater. Ind. Eng. Chem. Res..

[cit28] Supong A., Bhomick P. C., Baruah M., Pongener C., Sinha U. B., Sinha D. (2019). Adsorptive Removal of Bisphenol A by Biomass Activated Carbon and Insights into the Adsorption Mechanism through Density Functional Theory Calculations. Sustain. Chem. Pharm..

[cit29] Ahmad M., Moon D. H., Vithanage M., Koutsospyros A., Lee S. S., Yang J. E., Lee S. E., Jeon C., Ok Y. S. (2014). Production and Use of Biochar from Buffalo-Weed (Ambrosia Trifida L.) for Trichloroethylene Removal from Water. J. Chem. Technol. Biotechnol..

[cit30] Tiotsop Kuete I. H., Tchuifon Tchuifon R. D., Bopda A., Sadeu Ngakou C., Nche G. N. A., Gabche
Anagho S. (2022). Adsorption of Indigo Carmine onto Chemically Activated Carbons Derived from the Cameroonian Agricultural Waste Garcinia Cola Nut Shells and Desorption Studies. J. Chem..

[cit31] Huang Y., Li S., Chen J., Zhang X., Chen Y. (2014). Adsorption of Pb(II) on Mesoporous Activated Carbons Fabricated from Water Hyacinth Using H 3 PO 4 Activation: Adsorption Capacity, Kinetic and Isotherm Studies. Appl. Surf. Sci..

[cit32] Keiluweit M., Nico P. S., Johnson M. G., Kleber M. (2010). Dynamic Molecular Structure of Plant Biomass-Derived Black Carbon (Biochar). Environ. Sci. Technol..

[cit33] Ji L., Chen W., Zheng S., Xu Z., Zhu D. (2009). Adsorption of Sulfonamide Antibiotics to Multiwalled Carbon Nanotubes. Langmuir.

[cit34] Vidovix T. B., Januário E. F. D., Bergamasco R., Vieira A. M. S. (2021). Bisfenol A Adsorption Using a Low-Cost Adsorbent Prepared from Residues of Babassu Coconut Peels. Environ. Technol..

[cit35] Balci B., Erkurt F. E. (2017). Adsorption of Bisphenol-A by Eucalyptus Bark/Magnetite Composite: Modeling the Effect of Some Independent Parameters by Multiple Linear Regression. Adsorpt. Sci. Technol..

[cit36] Jaafarzadeh N., Baboli Z., Noorimotlagh Z., Silva Martínez S., Ahmadi M., Alavi S., Mirzaee S. A. (2019). Efficient Adsorption of Bisphenol a from Aqueous Solutions Using Low-Cost Activated Carbons Produced from Natural and Synthetic Carbonaceous Materials. Desalination Water Treat..

[cit37] Foo K. Y., Hameed B. H. (2010). Insights into the Modeling of Adsorption Isotherm Systems. Chem. Eng. J..

[cit38] Al-Ghouti M. A., Da’ana D. A. (2020). Guidelines for the Use and Interpretation of Adsorption Isotherm Models: A Review. J. Hazard Mater..

[cit39] Nworie F. S., Nwabue F. I., Oti W., Mbam A. E., Nwali B. U. (2019). Removal of Methylene Blue from Aqueous Solution Using Activated Rice Husk Biochar: Adsorption Isotherms, Kinetics and Error Analysis. J. Chil. Chem. Soc..

[cit40] Ashiq A., Sarkar B., Adassooriya N., Walpita J., Rajapaksha A. U., Ok Y. S., Vithanage M. (2019). Sorption Process of Municipal Solid Waste Biochar-Montmorillonite Composite for Ciprofloxacin Removal in Aqueous Media. Chemosphere.

[cit41] Ajala O. A., Akinnawo S. O., Bamisaye A., Adedipe D. T., Adesina M. O., Okon-Akan O. A., Adebusuyi T. A., Ojedokun A. T., Adegoke K. A., Bello O. S. (2023). Adsorptive Removal of Antibiotic Pollutants from Wastewater Using Biomass/Biochar-Based Adsorbents. RSC Adv..

[cit42] Weiss J. N. (1997). The Hill Equation Revisited: Uses and Misuses. FASEB J..

[cit43] Balarak D., Mostafapour F. K., Lee S. M., Jeon C. (2019). Adsorption of Bisphenol A Using Dried Rice Husk: Equilibrium, Kinetic and Thermodynamic Studies. Appl. Chem. Eng..

[cit44] Hilal N., Ahmed I., Badr E. E. (2012). Removal of Acid Dye (AR37) by Adsorption onto Potatoes and Egg Husk: A Comparative Study. J. Am. Sci..

[cit45] Ponnuchamy M., Kapoor A., Jacob M. M., Awasthi A., Mukhopadhyay M., Nandagobu S., Raghav A., Arvind D., Chakraborty P., Prabhakar S. (2025). Adsorptive Removal of Endocrine Disruptor Bisphenol A from Aqueous Environment Using Sugarcane Bagasse Derived Biochar. J. Taiwan Inst. Chem. Eng..

[cit46] Din S. U., Ngueagn P. T., Al-Ahmary K. M., AlMohamadi H., Al-Mhyawi S. R., Elamin N. Y., Alshdoukhi I. F., Alrashood J. S., Ofudje E. A. (2025). Adsorption of Bisphenol-A by Banana Biochar: Kinetic, Isotherms and Thermodynamics. Sci. Rep..

[cit47] Lingamdinne L. P., Angaru G. K. R., Pal C. A., Koduru J. R., Karri R. R., Mubarak N. M., Chang Y. Y. (2024). Insights into Kinetics, Thermodynamics, and Mechanisms of Chemically Activated Sunflower Stem Biochar for Removal of Phenol and Bisphenol-A from Wastewater. Sci. Rep..

[cit48] Shi W., Wang H., Yan J., Shan L., Quan G., Pan X., Cui L. (2022). Wheat Straw Derived Biochar with Hierarchically Porous Structure for Bisphenol A Removal: Preparation, Characterization, and Adsorption Properties. Sep. Purif. Technol..

[cit49] Leng L., Xiong Q., Yang L., Li H., Zhou Y., Zhang W., Jiang S., Li H., HUang H. (2021). An overview on engineering the surface area and porosity of biochar. Sci. Total Environ..

[cit50] Skic K., Adamczuk A., Gryta A., Boguta P., Tóth T., Jozefaciuk G. (2024). Surface areas and adsorption energies of biochars estimated from nitrogen and water vapour adsorption isotherms. Sci. Rep..

